# Healthiness of Food and Beverages for Sale at Two Public Hospitals in New South Wales, Australia

**DOI:** 10.3390/nu10020216

**Published:** 2018-02-15

**Authors:** Carrie Tsai, Erika Svensen, Victoria M. Flood, Yasmine Probst, Kathryn Reilly, Stephen Corbett, Jason H. Y. Wu

**Affiliations:** 1 Faculty of Dentistry, University of Sydney, Westmead, NSW 2145, Australia; 2 Faculty of Science, Medicine & Health, University of Wollongong, Wollongong, NSW 2522, Australia; eas076@uowmail.edu.au (E.S.); yasmine@uow.edu.au (Y.P.); 3 Western Sydney Local Health District, Westmead, NSW 2145, Australia; vicki.flood@sydney.edu.au (V.M.F.); stephen.corbett@health.nsw.gov.au (S.C.); 4 Faculty of Health Sciences, University of Sydney, Lidcombe, NSW 2141, Australia; 5 Illawarra Health and Medical Research Institute, Wollongong, NSW 2522, Australia; kathryn.reilly@hnehealth.nsw.gov.au; 6 Hunter New England Population Health, Wallsend, NSW 2287, Australia; 7 The George Institute for Global Health, University of New South Wales, Newtown, NSW 2042, Australia; jwu1@georgeinstitute.org.au

**Keywords:** food environment, nutrition guidelines, health facilities, food policy, marketing, hospitals

## Abstract

(1) Background: Our aim was to conduct objective, baseline food environment audits of two major western Sydney public hospitals and compare them to recently revised state nutritional guidelines. (2) Methods: A cross-sectional assessment was conducted (June–July2017) across 14 fixed food outlets and 70 vending machines in two hospitals using an audit tool designed to assess the guideline’s key food environment parameters of availability, placement, and promotion of ‘Everyday’ (healthy) and ‘Occasional’ (less healthy) products. (3) Results: Availability: Overall, Everyday products made up 51% and 44% of all products available at the two hospitals. Only 1/14 (7%) fixed outlets and 16/70 (23%) vending machines met the guideline’s availability benchmarks of ≥75% Everyday food and beverages. Proportion of Everyday products differed among different types of food outlets (café, cafeteria, convenience stores). Placement: On average, food outlets did not meet recommendations of limiting Occasional products in prominent positions, with checkout areas and countertops displaying over 60% Occasional items. Promotion: Over two-thirds of meal deals at both hospitals included Occasional products. (4) Conclusion: Baseline audit results show that substantial improvements in availability, placement, and promotion can be made at these public hospitals to meet the nutrition guidelines. Audits of other NSW hospitals using the developed tool are needed to investigate similarities and differences in food environment between sites. These findings highlight the need for ongoing tracking to inform whether the revised guidelines are leading to improved food environments in health facilities.

## 1. Introduction

Poor dietary behaviours are major drivers of chronic diseases globally. Australia is no exception—while only 5.1% of adults are meeting Australian Dietary Guidelines (ADG) for intakes of fruit and vegetables, 35% of daily energy is also coming from discretionary foods sources [1,2]. Such low compliance with Dietary Guidelines likely contributes to the high rates of obesity and chronic diseases in Australia, and strategies to improve conformity with Guidelines for Australians have been identified as a public health priority [3,4]. 

There is increasing recognition that individual dietary behaviours are shaped by key parameters of the food environment. For example, the relative availability of healthy versus less healthy foods and drinks, as well as the pricing and marketing of products, strongly influence consumer decisions [5]. Accordingly, there is growing acknowledgment that public sector institutions such as health facilities and schools should provide leadership in ensuring consumers are exposed to healthy food environments. Further, it follows that health facilities should prioritise healthy food environments that are consistent with healthy eating messages being provided by health professionals [6,7]. Health facilities can have a substantial impact, as they are frequented by large numbers of people. Australia’s public hospital system employs approximately 360,000 full-time equivalent staff and has over 6 million patient admissions annually [8]. 

Several Australian states recognise the importance and potential benefits of ensuring healthy food environments in health facilities and have developed food and beverage policies that aim to classify products (as healthy vs. less healthy) and define key marketing aspects of the food environment that these sites should aim to incorporate. Yet, very little robust research has assessed the prevailing food environments in health facilities in Australia in relation to such policies. This gap in knowledge makes it unclear if existing policies are achieving their objective to enable healthier food environments and, further, what specific areas might need to be targeted for improvements. 

The aim of this study was, therefore, to conduct cross sectional objective audits of the food and drink environments of the two largest hospitals in the Western Sydney Local Health District (WSLHD) to inform the ongoing implementation of the recently launched the Healthy Food and Drink in NSW Health Facilities for Staff and Visitors Framework (from here on, the Framework).

## 2. Materials and Methods 

### 2.1. Policy Context

The Framework, released in June 2017 [9], replaced the mandatory policy directive, Live Life Well @ Health: Healthier Foods & Drink Choices—Staff & Visitors in NSW Health Facilities [10], released in December 2009. One of the prominent updates was a move away from a traffic light-based food classification system (red, amber, and green designating healthfulness of a product) to a binary Everyday and Occasional food classification system. Everyday items consist of foods and drinks in the five core food groups based on the ADG [11], while Occasional items are considered ‘discretionary’ foods and drinks that are generally high in saturated fat, added sugars, and/or salt, such as crumbed/coated or processed meat, hot chips/potato wedges, cakes/muffins, confectionery, and diet beverages.

The aim of the Framework is to increase the supply and promotion of healthy foods and drinks to staff, visitors, and the general public in NSW health facilities and promote healthy choices as the easier choices. In-patient meals, foods, and drinks brought from home, and fundraising activities, are excluded. By setting targets for all food outlets, such as cafés or vending machines, to provide at least 75% Everyday foods and drinks, the Framework seeks to ensure that the consumer food environments in healthcare facilities contain a majority of healthy products. As the Framework provides additional guidance relating to maximum portion size of certain items and quality of ingredients, an additional Toolkit is available, which includes a ‘ready reckoner’ of Everyday and Occasional foods and drinks, as well as a ‘visual portion size guide.’ The Framework also sets marketing benchmarks, including promotion of only healthy products, pricing strategies to encourage the purchase of healthier products, and placement of only healthy products at key sales points. 

The Framework uses the Health Star Rating (HSR) system to guide food vendor choices towards healthier versions of some foods and drinks. HSR is a front-of-pack labeling system for packaged foods developed by the Australian State and Territory governments in collaboration with food industry, public health, and consumer groups, and is endorsed by the Australian Federal government. The nutritional profile of packaged food is rated from 0.5 to 5 stars in half star increments, with more stars indicative of healthier choices within a product category [12]. Within the Framework, a cut-off of 3.5 health stars or above is applied to certain packaged foods.

Additionally, the Framework calls for the removal of sugar-sweetened beverages (SSBs) from all outlets by December 2017, with compliance with other food-related requirements of the Framework to be addressed by December 2018. 

The Framework is based on the Australian Dietary Guidelines. We recognize that there is on-going debate related to the healthiness of different food and drinks, and there is a diversity of opinion related to the classification of certain categories of products. It is not in the scope of this study to provide in-depth review or to critique the classification system underpinning the Framework but rather, the current investigation aims to address how food and drink provision at these two public hospitals at baseline compares to the guidelines outlined in the Framework.

### 2.2. Design

A cross-sectional audit was conducted for all food outlets and vending machines in the two selected hospitals by six researchers. A paper-based audit tool was developed to assess the following Framework parameters: product availability, product placement, ‘promotional’ pricing (i.e., meal deals), and advertising of foods and beverages (Supplemental Materials, document S1). The design of the audit tool was informed by a review of existing measures for assessing food environments [5], as well as a previous tool used for vending machines [13,14]. The final audit tool was pre-tested (*n* = 3) for feasibility and amended to resolve ambiguities. To optimise reliability amongst researchers, a set of auditing protocols, such as counting procedures and accepted assumptions (Supplemental Materials, documents S2 and S3) from previous research, was modified and adopted [15]. 

While the Framework applies to pharmacies and florists, these were considered outside of the scope of this study due to the limited foods and beverages sold in these outlets and their focus on other products. Catering services for meetings and conferences were also considered outside of the scope of this study, because these services do not offer food and beverages to the general public at the hospital sites. 

Ethical approval was obtained from the WSLHD Human Research Ethics Committee (LNR/16/WMEAD/508).

### 2.3. Geographic Context

This study was based in Western Sydney, as it is one of the fastest growing regions in NSW, Australia [16] whose population has a higher risk of type 2 diabetes relative to other regions of NSW [17]. WSLHD includes four hospitals and seven community health centres. The two largest hospitals in the WSLHD were selected as a purposive sample for this study and have over 500 in-patient beds in Hospital A and over 400 beds in Hospital B [18]. At each site, one distinct commercial vendor provides the majority of non-vending machine food and beverages. There is no known affiliation between the two vendors. Subleases exist at both hospitals for a number of independent food outlets. A single vendor operates vending machines across both sites under site-specific contracts.

### 2.4. Data Collection

Data collection occurred between late June and early July 2017, in parallel with the release of the Framework in early June 2017. Table 1 outlines the types of food outlets. Two researchers conducted independent audits of each fixed food outlet using the audit tool. Audits were completed at the same time as per standard auditing practice to minimise variations in observations due to product turnover. Menus, product signage, promotions, and all food displays, including all products, were photographed by each researcher at the time of each audit. Following completion of each fixed food outlet, the two researchers compared outcomes, and where disagreement in count or categorisation occurred, a consensus was reached using the photographs and additional inspection or consultation with a supervising researcher (CT). Food outlet staff were asked for ingredient lists and portion sizes when these were not displayed. Where possible, the Framework’s visual portion size guide was used to determine compliance to the Framework guidelines on maximum portion sizes [9]. When the visual guide did not apply or actual serving was ambiguous, a single serve of the product was purchased and weighed using a Beurer KS48 (Beurer GmbH, Ulm, Germany) digital scale. Further clarification was sought from representatives of the NSW Ministry of Health when classification of specific food types was unclear in the Framework [19]. 

For vending machines, one researcher took photos of all vending machines on the same day as the fixed food outlet audits. The audit tool was then filled out by two researchers and confirmed by the supervising researcher (CT) based on the photos at a later date.

### 2.5. Study Outcomes 

Audited data were collated and analysed to compare the food environments at the two hospitals against the key food environment parameters in the NSW Framework as outlined below:

#### 2.5.1. Product Availability

In accordance with the Framework, all food and drinks were classified into Everyday or Occasional product categories. The percentage of Everyday products within all products (both Everyday and Occasional) was calculated for all food outlets.

An outlet was considered to meet the benchmark if at least 75% of total products displayed for sale were classified as Everyday products [9]. A second product availability outcome was also assessed, which was the percentage of sugary drinks available within all occasional drinks. The results from each hospital were reported separately, as both sites had different vendors and one had implemented the removal of sugary drinks before release of the Framework.

#### 2.5.2. Portion Size and Product Quality

Products were further classified as compliant or non-compliant with regard to portion size and ingredient quality, as the Framework provides additional guidance within Everyday and Occasional categories relating to these characteristics [9]. Food and drinks were reported separately within five categories:

- Compliant Everyday or Occasional food and drinks: met the maximum portion size and quality guidelines.
- Everyday food and drinks exceeding portion size (e.g., >400 mL fruit juice or >500 mL flavoured milk).
- Below quality Everyday food and drinks: contained “do not use” ingredients as identified from the product ingredient lists (e.g., muesli bars and nut mixes with added confectionary).
- Occasional food and drinks exceeding portion size (e.g., >280 g meat pie or >500 mL diet drink)
- Below quality Occasional food and drinks: contained “do not use” ingredients (e.g., hot meal with sour cream or any sugary drink, such as soft drinks, fruit drinks, sweetened iced teas, and energy and sports drinks) [9].

To avoid duplication, where an Everyday product both exceeded the portion size and did not meet the quality requirements (e.g., an oversized Everyday snack containing confectionary), the product was classified as below quality Everyday, regardless of portion size. Similarly, when an Occasional drink exceeded the recommended portion size and was classified as a sugary drink (e.g., a sugary drink >500 mL), this product was classified under sugary drinks. 

#### 2.5.3. Packaged Product Quality 

The quality of all applicable packaged food and drinks (per the Framework) were assessed using the HSR. The primary outcome was the percentage of packaged foods (within types of products for which HSR apply, per the Framework) that had a HSR ≥3.5. A list of these applicable product types can be found in the Framework [9]. Since the HSR system is currently implemented on a voluntary basis by the food industry (i.e., it is not currently found on all packaged products) [20], HSRs were identified from (1) front of pack label where available, or (2) the FoodSwitch database. The FoodSwitch database is developed by the George Institute from the collection nutrient information and calculation of HSRs as previously described [21,22]. 

#### 2.5.4. Placement and Promotion

Two prominent sales locations were assessed for presence of Occasional products: (1) checkout areas and, (2) countertops (granted they displayed food or drink products). The outcome assessed was proportion of respective these sites that contained any Occasional products.

Similarly, the degree of product promotion was assessed by advertisements and pricing strategies. As such, information was collected on the proportion of posters or counter displays and discounted meal deals that contained any Occasional products. 

### 2.6. Data Analysis

Audit data were managed with Microsoft Excel (v 14.4.0, Microsoft Corporation, Redmond, WA, USA, 2011) and SSPS Statistics (v 23, IBM Corp, Armonk, NY, USA, 2015). Descriptive statistics were calculated including the proportion and ranges of the food and drinks available at each health facility and food outlet. Differences in the proportion of Everyday food and drinks between the food outlet types were analysed using the Kruskal-Wallis or Mann-Whitney U-tests. Two sided p-values of <0.05 were considered significant.

## 3. Results

The two hospital sites included 84 food outlets in total (14 fixed food outlets and 70 vending machines). This study completed an audit of the 11 food outlets at Hospital A and 3 at Hospital B. Within Hospital A, one large ‘food court’ section was separated into 5 distinct food outlets, with each defined due to the presence of a checkout area. Forty vending machines were located at Hospital A, and another 30 at Hospital B.

### 3.1. Availability

**Figure 1:**
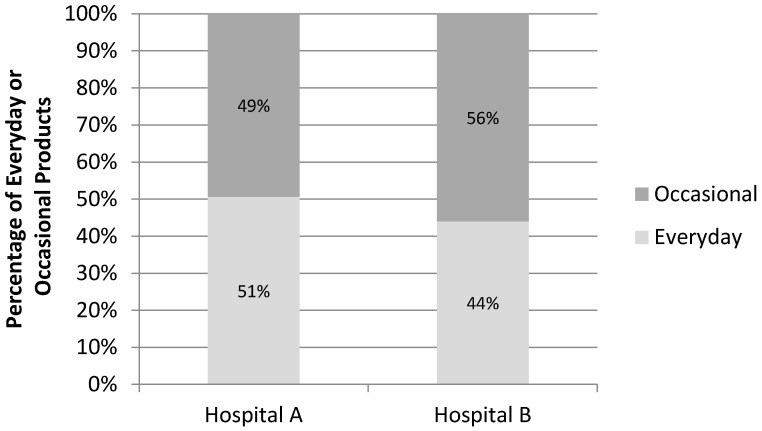
Proportion of Everyday and Occasional products for the two hospitals audited. Hospitals A and B had 2940 and 1783 total food and drink items available, respectively.

At the time of the audit, neither hospital met the Framework’s target benchmark of ≥75% Everyday products when considering all food and drinks combined (Figure 1). 

When assessed by type of food outlet, only one out of 14 (7%) fixed food outlets reached the benchmark. When food and drinks were considered separately, one out of 14 (7%) met the benchmark for food, while six out of 14 (43%) met the benchmark for drinks. No vending machines met the benchmark for food items alone, but 16 out of 70 (23%) of machines met the benchmark for beverages alone (Table 2). The proportion of fixed outlets and vending machines that met the availability benchmark appeared generally comparable between the hospitals.

The availability of Everyday products appeared to differ across the type of food outlets, an effect that was observed for both food and drinks (Table 3). For fixed food outlets, the café and cafeteria-type outlets had higher proportions of Everyday foods across all outlets (median 54%), compared to convenience stores (median 14%). Similarly, whereas cafés had the highest proportion of Everyday drinks (median 86%), convenience stores had substantially fewer Everyday drinks available (median 30%). 

For different types of vending machines, food-only machines fell below the benchmark at 23% (range 13%–54%) Everyday products, while drink-only machines were closer to meeting the benchmark at 70% (range 50%–100%) Everyday products. Of the eight mixed-vending machines, 33% (range 31%–15%) of drinks and 50% (range 42%–65%) of food items were classified as Everyday products (Table 3).

### 3.2. Product Quality

**Figure 2:**
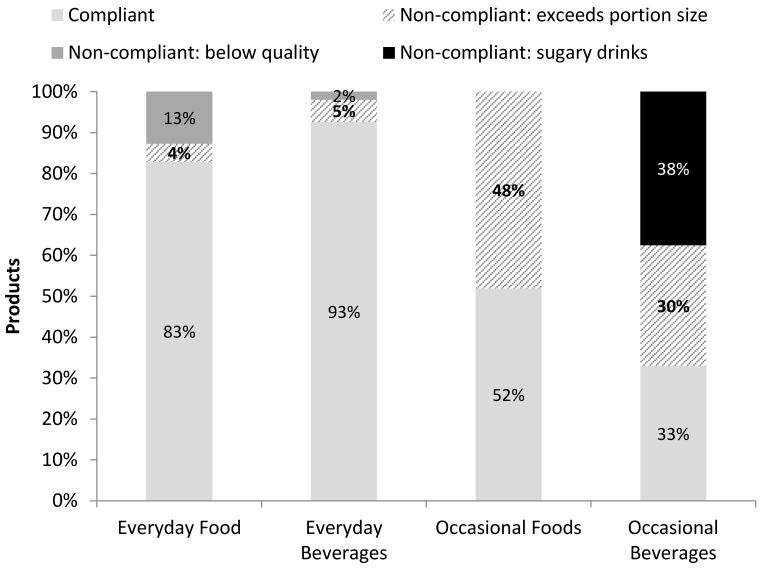
Proportions of products in categories based on portion size and quality of ingredients, per the Framework.

For Everyday foods, 13% (*n* = 97) were considered non-compliant due to Occasional ingredients (such as snack bars or nut mixes with confectionery). For Everyday drinks, 2% (*n* = 28) were considered as non-compliant, because they contained Occasional ingredients such as coffee drinks with whipped cream (Figure 2). Sugary drinks made up 38% (*n* = 360) of all Occasional drinks. When assessed by hospital, 70% of Occasional drinks in Hospital B were sugary drinks, compared to 7% at Hospital A (Appendix, Table A1).

Health Star Ratings (HSR) applied to 147 packaged food and 77 beverage items. Incidentally, only 21 (14%) of food and 4 (5%) of drink items had HSR on front-of-package. Fourty (27%) and 23 (30%) of total applicable packaged food and drink items, respectively, were not found in the database, nor had front of package HSR. Of the remaining products, 41% (*n* = 44) of food and 59% (*n* = 32) of beverages had ≥3.5 HSR.

### 3.3. Product Size 

A small proportion (4%, *n* = 34) of all Everyday foods exceeded portion size limits, most of which were lightly salted nuts/seeds, dried fruit, or snack bars that exceeded 50 g. About 5% (*n* = 80) of all Everyday drinks exceeded portion size limits, most of which were diet soft drinks or flavoured milk products that exceeded 500 mL (Figure 2). Not all Everyday foods had portion size limits per the Framework. When assessing products that did have this restriction, 46% of Everyday foods and 21% of Everyday drinks exceeded potion size limits (Appendix, Table A2).

For Occasional foods, 48% (*n* = 730) had portion sizes exceeding those specified by the Framework. A substantial proportion (30%, *n* = 283) of Occasional drinks also exceeded portion size limits. Of note 46% (*n* = 167) of sugary drinks were oversized. 

### 3.4. Placement and Promotion

Of all fixed outlets, a large proportion of prominent sales areas contained Occasional food or drinks including nine out of 14 (64%) checkout areas and six out of nine (67%) countertops that contained items. Twenty-eight promotional posters were observed across all fixed outlets, among which 12 (43%) promoted Occasional products. Finally, six out of nine (67%) of all meal deals were observed during the audit and included Occasional products (Table 4).

Overall, a small number of fixed outlets (*n* = 3, 21%) met the marketing benchmark for the placement of only Everyday products in prominent areas. Similarly, only three of the outlets (21%) actively promoted only Everyday products (Table 4). 

## 4. Discussion

The audit, which was conducted shortly after the release the Framework, provides baseline data on the type and level of changes to the food environment that will be needed to meet the target to have healthier food choices in place by December 2018. With regard to product availability, the proportion of Everyday products was found to be around 50% at both hospitals. Only 7% of fixed food outlets and about 20% of vending machines met the benchmark of ≥75% Everyday products. Our findings also highlight other key aspects that could be improved in relation to product quality, portion size limits, placement, and promotion practices. 

To date, few studies have conducted objective audits to assess the healthfulness of the hospital consumer food environment in Australia. A previous study in the NSW Hunter New England Health District audited product availability at five food outlets and 114 vending machines using the previous NSW guidelines for health facilities. As their results were based on the ‘traffic light’ food classification system, findings are not directly comparable to the present study. Nevertheless, this previous study also reported a large proportion of food and drinks available were unhealthy products (classified as red), particularly for vending machines [6]. This current study builds upon the previous study, as we assessed findings against the updated NSW food procurement guidelines for health facilities and investigated additional food environment parameters. Studies in the United States and Canada have also reported that low proportions of healthy products are available in hospital food outlets [23,24,25,26]. Together, these studies and our findings suggest strategies to improve the relative availability of healthier products, in accordance with government healthy food and drink guidelines for health facilities, are urgently needed.

In Australia and other countries, portion sizes of many discretionary foods have increased substantially over the last few decades [27,28,29]. A growing body of literature suggests that increased portion size is likely to contribute to increased energy intakes and the obesity epidemic, particularly for older children and adults [30,31]. Consistent with trends of increasing portion sizes for other food service settings, our findings suggest that a substantial amount of discretionary products available in Australian hospitals currently exceed government recommendations. This was particularly alarming for sugary drinks, over-consumption of which is linked to dental decay, obesity, and type 2 diabetes. Controlling the portion size of discretionary food and drinks has been identified as an important aspect of the food environment that could lead to improved dietary consumption [32,33], with modeling suggesting that limiting portion size of discretionary products is likely to bring significant health benefits [34]. Further, the use of policy to curb portion sizes in large institutions could also motivate manufacturers to offer smaller portions and assist in resetting consumer norms [35]. 

In addition to exceeding portion size recommendations, a small proportion of Everyday products contained discretionary ingredients and therefore did not meet the Framework quality criteria. These categories of food and drinks, like snack bars and mixed nuts that contained confectionery or coffee drinks with whipped cream, could be an immediate area that food vendors could improve, as ‘compliant’ versions of these types of products exist and could be substituted. Strategies to help meet the requirements of the Framework may include education of food vendors through aids such as product lists that meet quality of ingredient criteria.

A key government-led approach to improving food environments is front-of-package labelling, as packaged foods are an increasingly important part of the food supply. Front-of-package nutrition labels can offer guidance to consumers on the healthfulness of the food product in a separate fashion than nutrition information panels that are often harder to see and difficult to understand. Our findings based on the FoodSwitch database suggest many packaged food and drinks available in the audited health facilities have sub-optimal nutrient profile (<3.5 Health Star Rating [HSR]). However, our results also highlight that currently less than one in six of applicable packaged products actually carried a front-of-package HSR. This presents an obvious obstacle for food vendors to identify healthier packaged food products. Moving forward, the policy alignment between NSW and the federally-run HSR system could be significantly improved by making the HSR a mandatory requirement on all packaged foods [12,20]. In the interim, searchable food and drink databases with HSRs, such as FoodSwitch and one being created by the Ministry of Health, are available to vendors in local health districts [19,21].

Marketing research has shown how point of purchase promotion and placing products at other eye-catching and often high-traffic positions increases sales [36,37,38]. Our findings highlight that these strategies are being used in health facilities to promote less healthy products. Interestingly, recent studies suggest changing such promotional practices can be achieved without impacting the overall sale of food outlets. For example, a study in a self-service café in an Australian health facility trialed the removal of less healthy, red traffic light-classified drinks from main fridges and placed them out of sight behind the counter. After six weeks, the intervention reduced the proportion of ‘red’ drinks sold from 33% to 10% of total drinks sold, with a concomitant significant increase in healthier ‘amber’ and ‘green’ traffic light-classified drinks. Overall sales volume was unchanged and the retailer chose to continue with the strategy after the trial period [39]. Additionally, there is evidence that consumers of hospital food and drinks outlets would prefer the provision of healthier food items, based on a survey with a focus on vending machines of people attending a hospital in regional NSW [40]. 

Existing literature suggests that implementation assistance including training and ongoing support are predictors of compliance with government healthy food and drink policies in primary and secondary schools [41]. Similarly, organisational leadership has been shown to be vital for policy implementations [42] such as when key executive stakeholders place health high up in organisational priorities. During the period of our study, one of the hospitals demonstrated leadership in implementing a restriction on the sale of sugary drinks well before the NSW Framework timeframe (in March 2017). To achieve this, the team secured buy-in from senior executives, relevant hospital departments, and retailers, who are seen as partners in implementation of the Framework. Importantly, a comprehensive communication strategy targeting staff, visitors, and patients was also developed and implemented [9]. Our findings, which reflected this removal of sugary drinks in one of the hospitals, support the feasibility of phasing out sugary drinks with a well-supported implementation plan, although ongoing monitoring is needed to ensure compliance is maintained.

Another commonly reported barrier is the difficulty in sourcing healthier items in certain settings, such as convenience stores or for food vending machines [43,44,45,46]. Our results support these prior observations, since at the two hospitals audited, the proportions of Everyday products in convenience stores and food vending machines were among the lowest proportions observed. Thus, these settings may require extra support as they tend to offer a small range of foods that are predominantly packaged. A possible support strategy is the development of ‘buyers guides’ that list healthy alternatives to commonly sold items, such as those that are already available for schools [47]. 

### Strengths and Limitations

We conducted a detailed and objective audit that eliminated self-report bias and provided a detailed assessment of key parameters of the food environment. We also consulted directly with NSW Health, designers of the Framework, to ensure accuracy of product classification. Our detailed audit complements efforts by policy makers in NSW to use a ‘practice-based’ approach that will target and monitor a set of high-level key practices [19]. Going forward, both approaches are needed to ensure improvements in the hospital food environment are made over time. Another strength was our ability to assess healthiness of packaged foods using the FoodSwitch database, which provides HSRs that are currently not available on most packaged foods. 

While we cannot exclude the possibility that some products may have been misclassified, our audit was designed to minimise such error through a number of procedures: audit training including a detailed procedure manual for each researcher, pilot testing prior to the audit conducted by all researchers, and lastly, a consensus meeting after each site audit which reached 100% consensus in all sites. The study was limited to two health facilities in one local health district, and the findings may not be generalisable to other hospitals in NSW or other jurisdictions in Australia. Additionally, the use of single point data collection has the potential to limit the findings by an inability to capture seasonal changes in stock or promotions [5]. Nevertheless, our findings are consistent with previous audits in Australia and other countries that also suggest less healthy food environments in hospitals. 

The findings of this audit should be considered as a baseline for future repeat audits of the same facilities to determine the success of implementation of the NSW Framework with an aim to expand the audited sites to other hospitals in different health districts for comparability.

## 5. Conclusions

Baseline audit results of these public hospitals show that substantial improvements can be made in the main parameters of the food environment to meet nutrition guidelines—namely, availability, placement, and promotion. Audits of other NSW hospitals using the developed tool are needed to investigate similarities and differences in food environment between sites. These findings highlight the need for ongoing tracking to inform whether the revised guidelines and implementation support are leading to improved food environments in health facilities.

## Figures and Tables

**Table 1:** Types and number of food outlets audited.

Café (*n* = 7)	Sells freshly prepared hot beverages and may sell a selection of pre-made fresh food products
Cafeteria (*n* = 5)	Sells either hot or cold fresh food products served to order, may also have a selection of premade food
Convenience store (*n* = 2)	Majority of items sold are manufacturer packaged products, limited fresh options available
Vending machine (*n* = 70)	Three types of vending machines were observed: food only, beverage only, or containing both food and beverage

**Table 2:** Number of outlets meeting the Framework benchmark of ≥75% Everyday food and drinks.

	No. of Outlets	≥75% of Total Everyday Products, *n* (%)	≥75% Everyday Food, *n* (%)	≥75% Everyday Drinks, *n* (%)
**Hospitals Combined**				
Fixed outlets	14	1 (7)	1 (7)	6 (43)
Vending machines	70	16 (23)	0	16 (23)
**Hospital A**				
Fixed outlets	11	1 (9)	1 (9)	5 (45)
Vending machines	40	10 (25)	0	10 (25)
**Hospital B**				
Fixed outlets	3	0	0	1 (33)
Vending machines	30	6 (20)	0	6 (20)

**Table 3:** The proportion of Everyday food and beverage products by types of outlet type across the audited hospitals combined.

Outlet Type (n)	Food	Beverage
	% Everyday Median (Range)	% Everyday Median (Range)
**Fixed Outlet ^†^**		
Café (7)	54 (23–67)	86 (64–92)
Cafeteria (5)	54 (20–82)	54 (45–64)
Convenience store (2)	14 (5–22)	30 (16–44)
	*p* = 0.132	*p* = 0.005
**Vending machines ^§^**		
Beverage vending (39)	N/A	70 (50–100)
Mixed vending (8)	50 (42–65)	33 (31–35)
Food vending (23)	23 (13–54)	-
	*p* < 0.001	*p* < 0.001

^†^ Kruskal-Wallis H tests, ^§^ Mann-Whitney U-tests.

**Table 4:** Number of prominent areas, advertisements, and meal deals containing Occasional products.

	Product Placement n/N(%)		Advertisements * n/N(%)	Meal Deals n/N(%)
	Checkout Areas	Countertops		
Hospitals Combined	9/14 (64)	6/9 (67)	12/28 (43)	6/9 (67)
Hospital A	7/11 (64)	5/7 (71)	3/15 (20)	2/5 (40)
Hospital B	2/3 (67)	1/2 (50)	9/13 (69)	4/4 (100)

* The advertisements observed were wall posters or stand up advertisements near the check out areas.

## Supplementary Materials

The following are available online at http://www.mdpi.com/2072-6643/10/2/216/s1, Document S1: Food Environment Audit Tool, Document S2: Counting Procedure, Document S3: Food Audit assumptions.

## Appendix A

**Table A1:** Proportion of all Occasional drinks that are sugary drinks, by hospital.

	Sugary Beverages	All Occasional Beverages	Proportion Sugary Beverages
Hospital A ^†^	35	492	7%
Hospital B	325	467	70%

^†^ Hospital A implemented a restriction on the sale of sugary beverages 9 months before the NSW Framework timeframe (in March 2017) and before the audit.

**Table A2:** Non-compliant products within the applicable products per the Framework.

	Products Exceeding Portion Size	Total	Proportion Exceeding Portion Size
Food ^†^	86	406	21%
Beverages ^§^	64	139	46%

^†^ Foods with maximum portion sizes are: packaged ready-to-eat meals (450 g); muesli and snack bars (50 g); dried fruit (50 g); lightly salted or flavoured popcorn, legumes, nuts, and seeds (50 g); sweet biscuits (50 g); cakes (80 g); sweet pastries (80 g); desserts (100 g); confectionery (50 g); ice-cream, frozen yoghurt, and ice-blocks(85 mL). ^§^ Drinks with maximum portion sizes are: fruit/vegetable juice (400 mL); flavoured milk, milkshakes/smoothies, liquid breakfast drinks and coffee (500 mL); diet drinks (500 mL).
