# Supplementary material for: Healthiness of Food and Beverages for Sale at Two Public Hospitals in New South Wales, Australia
Source: Nutrients. 2018 Feb 15;10(2):216. doi: 10.3390/nu10020216 (PMC5852792; doi:10.3390/nu10020216)
Supplement: Supplementary file 1 [file nutrients-10-00216-s001.zip › Document_S1(AuditTool).pdf]

Food Environment Audit Tool

**Health Facility:**		**Location:**
**Date audit completed**	**Time:**	**Initials of auditor:**
**Type of outlet /name**		
**Point of Sale** (describe what is available)	1) Checkout Areas	2) Countertops
**Marketing**	**Number of promotional posters at outlet** (describe what is displayed)	**Meal deals** (list any discounted meals that include occasional foods)

Category	List of Foods and Drinks	# types	Comments
Everyday Snacks	**Canned tuna/chicken** (plain, spring water, including packaged with plain crackers/ salads/beans/pasta. Does not include processed chicken roll/loaf)		
	**Cheese**		
	**Custard (no added confectionary)**		
	**Dips** (hommus, tzatziki, veg-based) NOT cream-cheese or sour-cream-based dips)		
	**Dried fruit** (non-coated) **(<50g)**		
	**Fruit** (if canned, in juice,not in syrup)		
	**Unsalted nuts, seeds, popcorn, legume snacks** (including mixed with plain dried fruit)		
	**Lightly salted nuts and seeds (<50g)** (no sweet coratings)		
	**Lightly salted and flavoured popcorn (<50g)** (including lightly salted microwave varieties), not coated with caramel, toffee, carob, chocolate)		
	**Lightly salted legume snacks (<50g)** (wasabi peas, roasted and salted chickpeas)		
	**Muesli and Snack bars (<50g) (**e.g. cereal-based bars, fresh or dried fruit-based bars, nut bars, protein bars and breakfast bars No added yoghurt or confectionery e.g. chocolate, chocolate chips, marshmallow, sprinkles)		
	**Savory biscuits/crackers/crispbread** (preferably whole grain)		
	**Scones, pikelets, pancakes** (no added confectionary)		

	**Vegetables & Legumes (lentils/beans)**		
	**Yoghurt** (greek, natural, plain, flavoured, soy, excludes coconut-milk based) No Added confectionary.		
Occasional Snacks	**Cakes & sweet pastries** (muffins, banana bread, brownie, carrot cake, choc cake, fruit cake, cupcake, croissants) **(<80g)**		
	**Confectionery** (choc, choc spreads, boiled sweets, fruit jellies, icing, yoghurt type confectionary or yoghurt coating) **(<50g)**		
	**Desserts** (mousse, cheesecake, puddings, rice pudding, custard, jelly) **(<100g)**		
	**Ice cream, frozen yoghurt, ice blocks (<85ml)**		
	**Salty snacks** (e.g. chips and crisps (potato, corn, grain and wholegrain-style, vegetable), flavoured baked savoury biscuits, extruded snacks (cheese, bacon, BBQ flavoured puffed snacks, extruded vegetable snacks), coated nuts, soy crisps, flavoured rice snacks, noodle snacks and pretzels (**<50g)**		
	**Sweet biscuits (<50g)**		
Everyday Cold Meals	**Frittata** (no pastry) (everyday fillings)		
	**Salads** (garden, pasta, bean, couscous, quinoa, egg, potato-based, coleslaw, tabouleh)		
	**Sandwiches/wraps/rolls (**everyday fillings, any type bread)		*lean ham is Everyday ingredient
	**Sushi/rice paper rolls -** everyday fillings (tuna, avocado, salmon, veg, tofu, chicken, uncoated lean meats) (NO deep fried/tempura)		

Occasional Cold Meals	**Sandwiches/wraps/rolls/salads/sushi/frittata** (occasional fillings eg bacon, ham, salami, chorizo, prosciutto, coated/crumbed meat eg chicken schnitzels, coated/crumbled falafel, vegetable tempura, food with pastry) **(<60g processed meats/<140g crumbed coated meats)**		
Everyday Hot Meals	**Dim sum, wontons, steamed buns** (not fried)		
	**Pasta (**lasagne, spaghetti bolognaise, ravioli, macaroni and cheese, pasta bakes, canned spaghetti)		
	**Pizza** (Everyday toppings e.g. roast chicken, veg, pineapples, cheese, preferably thin crust,muffin/pita base, and wholemeal)		
	**Risotto and rice dishes (**chicken risotto, mushroom risotto, vegetable risotto, nasi goreng, mexican rice, paella)		
	**Indian**		
	**Asian – with rice**		
	**Asian – with noodles**		
	**Mexican** (soft tortillas only)		
	**Soup**		
	**Jacket (baked) Potatoes -** everyday toppings (salad, tuna, baked beans, sweetcorn, savoury lean meat)		
	**Burger patties/rissoles/meatballs/falafel/veggie** (uncoated)		
	**Toasties and open melts** (everyday fillings)		
	**Everyday Hot Breakfast** (everyday fillings) *lean ham is Everyday, no deep fried food		

Occasional Hot Meals	**Hot meals with OCCASIONAL food/fillings (<450g)** (pizza/pasta/risotto with processed meats such as bacon/prosciutto; meals with crumbed/ coated meat, such as schnitzel meals or crumbed burgers; meals with hot chips/wedges; sausage sizzle or meals with sausages; breakfast or other meals with bacon/sausages/hash browns; nachos or tacos with corn chips or hard taco shells/bowls; jacket potato or burgers with processed meals such as bacon; meals including garlic bread or pastry) *lean ham is Everyday ingredient		
	**Crumbed and coated foods** (hot or cold) **(<140g)**		
	**Garlic bread & cheese/bacon rolls/pull-apart breads (<90g)**		
	**Hot potato products** (chips, hashbrowns, wedges, gems, scallops, potato bakes) **(<100g)**		
	**Instant flavoured noodles/ hot noodle cups** (<75g dry pack weight)		
	**Corn chips, hard taco shells/bowls-tacos/nachos/bowls (<50g)**		
	**Pies (<180g, <250g for potato top)**		
	**Processed meat** (bacon, chicken roll, pastrami, salami, pepperoni, chorizo, pancetta, prosciutto, mortadella, frankfurts, sausages and hot dogs. Does not include compressed whole muscle-meat products, such as formed chicken-breast, free-flow chicken, formed fish-fillet (<60g)		
	**Sausage rolls, savoury pastries** (sausage rolls, spring rolls, samosas, quiche, filled patries, cheese&spinach triangles) **(<120g)**		

Everyday drinks (not in fridges)	**Water** (tap, spring, mineral, sparkling) (includes infused with flavouring, fruit/herb essence or fruit juice flavouring up to 4%)		
	**Plain milk, milk alternatives**		
	**Tea (leaf/herbal)** No added sugars or intense sweeteners		
	**Fruit/vegetable juice (including coconut water)** (99% fruit or veg) (no added sugar or intense sweeteners) **(<400ml)**		
	**Coffee (freshly made)** (All espresso, instant, filtered, percolated coffees e.g. coffee made with water and/or milk, latte, cappuccino, mocha, and iced-coffee. May be blended with ice (frappe) or frozen. Preferably reduced fat. **(<500ml)**		
	**Liquid breakfast drinks/protein drinks (<500ml)**		
	**Flavoured milk/alternatives** (e.g. chocolate flavoured milk, iced-coffee flavoured milk, hot chocolate, iced chocolate, drinking yoghurt. Flavoured milk and milk alternatives served frozen or blended with ice (e.g. frappes) are Everyday drinks. **(<500ml)**		
	**Milkshakes/smoothies** (fruit based, or milk/milk/alternative-based, no ice-cream/gelato) **(<500ml))**		
Occasional drinks (not in fridges)	**Diet and sugar-free drinks** (e.g. diet soft drinks, diet iced teas, diet flavoured mineral waters, diet energy drinks, diet sports waters and sports drinks, diet cordials and no-sugar slushies) **(<500ml)**		
	**Thick shakes/smoothies** (w/ ice cream/gelato/sorbet) **(<500ml)**		

Drinks

**A**	Water (still, mineral, lightly flavoured, sparkling plain, no sugar added)	**<u>Please list any additional items in the vending machine, and which items were over the appropriate portion size:</u>**
**B**	Water (fortified) (eg, Vitamin or Smart Water)	
**C**	Lightly sparkling water (flavoured)	
**D**	Coconut water	
**E**	Milk, plain (or milk alternative)	
**F**	Milk, flavoured (<500ml)	
**G**	Fruit/Vegetable Juice (99%) (<400ml)	
**H**	Fruit drink	
**I**	Iced Tea	
**J**	Soft Drinks (non-diet e.g. Cola/Sprite/Lift/Fanta/Deep Spring) (<500ml)	
**K**	Diet soft drinks (e.g. Coca Cola Zero, Coke Life, Sprite Zero) (<500ml)	
**L**	Sports drinks (normal e.g. Powerade Ion)	
**M**	Sports drinks (sugar-free)	
**N**	Energy Drinks (normal)	
**O**	Energy Drinks (sugar-free)	
**P**	Protein drinks	
**Q**	Liquid breakfast drinks (<450g)	
**R**	Coffee flavoured drink	

Fridge One:

Commercial fridge? Yes/No

Shelf	Start from Left to Right; Item x Quantity in rows
1 (Top)	
2	
3	
4	
5	
6	

Fridge Two:

Commercial fridge? Yes/No

Shelf	Start from Left to Right ; Item x Quantity in rows
1 (Top)	
2	
3	
4	
5	
6	
