# Supplementary material for: Healthiness of Food and Beverages for Sale at Two Public Hospitals in New South Wales, Australia
Source: Nutrients. 2018 Feb 15;10(2):216. doi: 10.3390/nu10020216 (PMC5852792; doi:10.3390/nu10020216)
Supplement: Supplementary file 1 [file nutrients-10-00216-s001.zip › Document_S2(CountingProcedure).pdf]

# Counting Procedure

If an item is for purchase we will attempt to treat it as an 'individual item' and therefore assign it a count (regardless of whether this is repeated elsewhere). The updated Food and Drinks guidelines have different ways of assigning products and this needs to be taken into account when counting items.

**Types of Food and Drink displays:**

- **Packaged food displays**: To assess the proportion of Everyday foods and drinks in locations where only packaged foods and drinks are displayed, count the **number of facings**.

  -Packaged foods include products such as drinks in cans or bottles, packs of confectionery, chips, etc.
  -A facing is a food or drink positioned on the edge of a shelf or at the front of a cabinet facing the customer. Each facing is to be counted as one. For example, in the drinks fridge shown below, the top row has eight facings.

- **Buffet-style displays**: To assess the proportion of Everyday foods in display units where trays of bulk foods are displayed to be plated for, or by, the customer, count the **individual food types**, rather than the facings.

  - This type of display includes salad bars, and hot food displays serving, for example, lasagne, stir fry, roast meat and vegetables, mashed potato, rice, hot chips.

- **Ready to order foods**: To assess the proportion of Everyday foods in display units where many types of freshly prepared foods are stocked and ready for purchase, count the **individual food types**, rather than the facings.

- **À la carte**: To assess the proportion of Everyday foods where food is ordered from a menu, rather than displayed, count the individual food types listed on the menu.

*Note: the NSW Framework designates that each of the following food outlets should have a 75% of Everyday foods/drinks*

- Drinks fridge
- Chilled food cabinet
- Hot food display
- Shelf unit
- Free standing unit

| Item | Counted as | Reasoning |
|---|---|---|
| **General counting situations** | | |
| Same Product- Different *Sizes* | Separate items | The serving size of a product can determine assessment category, therefore ALL serving sizes of the same product are counted as separate items e.g. small hot meal and large hot meal count as 2, wraps and half wraps count as 2, cup or plate of chips count as 2. |
| Same Product- Different *Flavours* | Separate items | Although flavourings would not typically change assessment category they do add to the number of items for purchase and therefore should be counted to account for this. |
| Product with number of flavours not specified | Count once | We cannot make an assumption on how many flavours are available.E.g. Up&Go w/no flavour information=1EVERYDAY item |
| Meal deals | Separate items **if not on menu elsewhere** | If the items have already appeared on the menu separately then don't count. However, if they haven't appeared on the menu then count separately according to category. |
| **Food items** | | |
| Sandwich/wraps types -menu, pre-made | Count once | First count sandwich/wrap types on menu, then other pre-made sandwiches if not on menu already. |
| Sandwich/wraps types -'choose your own' | Separate main items | For 'choose your own,' count each individual **main protein/ingredient** as separate items. For example, choose your own sandwich (1) with ham (1), falafel (1), hummus (1), salad (1) |
| Pizzas (including 'choose your own toppings') | Combined items | Count each type of purchasable pizza as a separate item |
| Bread varieties – white, wholemeal, multigrain etc. | Single item – count once | If sold as a separate item, count once (nutritional profile may be different but not relevant to the criteria), but NOT as a sandwich option |
| Sauce | Do not count | These cannot be assigned to a category (sauces have not been designated a category in the guidelines) |
| Cakes, muffins (various types or flavours) | Separate items | Although flavourings would not typically change product category they add to the number of occasional items available and therefore should be counted to account for this. |
| Fruit salad/canned/dried fruit/frozen fruit/fruit kebabs | Separate items | Count 1 item for each fruit category |
| Fruit basket: Apple, banana, orange | Separate items | Separate items, as consumers can choose each type separately. If menu only states "seasonal fruit" only count as 1 item |
| Pikelets or bagel (with and without spread) | Separate items | Count as 1 EVERYDAY item for each option. For example pikelets/bagel with jam (1), margarine (1) or plain (1), cream cheese (1).If spreads are sold separately they will not be counted as they cannot be assigned a category (spreads have not been designated a category in the guidelines) |
| Popcorn/chips/extruded products like Twisties | Separate items | Each snack variety and all flavours are counted as separate menu items |
| Muesli or snack bars | Separate items | Each snack variety, all flavours, and sizes are counted as separate menu items |
| **Drinks** | | |
| Coffee/tea/chai | Separate items | Separate menu items as they may be made differently |
| Milo, hot choc | Separate items | Separate menu items as milo has a different nutrient profile to hot chocolate |
| Hot/Cold Milo | Separate items | Separate menu items as they may be made differently (e.g. hot milo may have water added), be different sizes and/or price |
| Drinks, different sizes | Count once | Count 1 item per type of drink |
| Milk - Oak/Dare/Moove/Calciyum | Separate items | Each brand/variety would have a different nutrient profile therefore each brand/variety is counted as 1 separate item. |
| Extra syrups/flavourings | Do not count | These cannot be assigned to a category (no more than 1 level tablespoon of flavouring powder or 20mL syrup should be used per portion). |
