# Supplementary material for: Healthiness of Food and Beverages for Sale at Two Public Hospitals in New South Wales, Australia
Source: Nutrients. 2018 Feb 15;10(2):216. doi: 10.3390/nu10020216 (PMC5852792; doi:10.3390/nu10020216)
Supplement: Supplementary file 1 [file nutrients-10-00216-s001.zip › Document_S3(AuditAssumptions).pdf]

Food Audit Assumptions

1 Background

The Food Audit Assumptions outline the categorisation of food and drink items (Everyday, Everyday non-compliant, Occasional, Occasional non-compliant) when there is a lack of information provided by the food vendor or the item is not included in the Food and Drink Guidelines. These assumptions were developed to ensure consistency in food audits in health facilities.

Assumptions have been made accordingly for when an item where the product information is unknown (see Table 2: Lack of information from food vendor).

The assumptions are only to be used if no further information can be collected from food vendors and a consensus as to which category the product fits within cannot be reached by the team.

2 Assumptions

The following rule applies when menu items contain Occasional products.

- If a menu item contains the following Occasional ingredients, it automatically becomes **Occasional.** (Crumbed/coated meat, processed meat (except ham), hot chips)
  -For example, an egg and bacon roll, or tempura sushi would be assessed against Occasional criteria
  -However, confectionery in an Everyday food or drink does NOT make it Occasional, but makes it non-compliant and should never be sold.

The tables in the pages that follow outline the assumptions that are to be made to assist in determining the category of menu items.

Table 1 – Menu assessment key
Table 2 – Assumptions to use in the lack of information from food vendors

Table 1: Menu Analysis Key

Abbreviation	Category
E	Everyday
ENC	Everyday non-compliant
O	Occasional
ONC	Occasional non-compliant
HSR	Health Star Rating

Table 2: Assumptions to use in the lack of information from food vendors

Menu item	Freshly prepared on site/packaged	Assumed category	Reasoning
**Sandwiches/wraps/rolls/toasties**			
Fillings – lean meat, tuna, salmon, roast chicken, reduced-fat cheese, salad/ vegetables, egg, falafel	Freshly prepared/ packaged	**E**	Assume fillings are Everyday and have ≥3.5 health star rating (HSR)
Fillings – ham, bacon, sausages, coated/crumbed meat (e.g. Chicken schnitzel)	Packaged	**O**	Assume fillings are Occasional and have ≥3.5 HSR<br>For portion sizes assume:<br>• Processed meat (e.g. ham, bacon) <60g.<br>• Sausages < max 60g.<br>• Crumbed and coated meat (e.g. chicken tenders) <140g.
**Bread**			
Bread e.g. white, wholemeal, multi-grain, rye	Packaged	**E**	Assume all bread has ≥3.5 HSR therefore Everyday
Margarine on bread	Packaged	**E**	Assume margarine has ≥3.5 HSR
**Sushi and Salads**			
Sushi	Freshly prepared or packaged with lean meat e.g. tuna rolls, teriyaki chicken	**E**	Assume the following fillings are Everyday - Tuna, salmon, fresh chicken breast, avocado, vegetables or tofu.
	Freshly prepared or packaged with crumbed meat	**O**	Assume the following fillings are Occasional - crumbed meats, processed meats<br>**Note:** aligns with salad and processed meat assumption but not covered in guidelines
Salads containing 'non crumbed meat'	Freshly prepared/ packaged	**E**	Assume all salad ingredients are Everyday have ≥3.5 HSR and/or on the 'buy' section of the Essential shopping list
Salads containing crumbed meat eg. Chicken tender salad	Freshly prepared/ packaged	**O**	Assume Occasional if salad contains Occasional ingredients such as crumbed or processed meats (≥3.5 HSR).<br>Assume Salad ingredients are Everyday fresh unpackaged or Everyday packaged with ≥3.5 HSR and/or on the 'buy' section of the Essential Shopping List.
Caesar salad	Freshly prepared/ packaged	**E**	Assume Caesar salad contains Everyday packaged ingredients that have ≥3.5 HSR.
**Burgers**			
Burgers (uncoated meat, tofu, fish, beef, chicken, vegetable, lentil)	Freshly prepared/ packaged	**E**	Assume burger patties are made with Everyday ingredients (lean meat, vegetables or lentils) or packaged ingredients which have ≥3.5 HSR and/or foods from the Essential Shopping List.<br>If packaged burger patty, assume portion size < max
Burgers (crumbed or coated meat/falafel)	Freshly prepared/ packaged	**O**	Assume burger patties has ≥3.5 HSR
**Meat/fish/poultry and alternatives**			
Processed meat e.g. Ham, devon, salami,	Packaged	**O**	Check portion size and HSR label on FoodSwitch database

bacon, frankfurters, hot dogs			
Tuna, salmon, roast beef, skinless chicken, turkey	Packaged	**E**	Assume all lean meats are Everyday and have a ≥3.5 HSR
**Hot food**			
Pasta	Freshly prepared (tomato, mince based or cheese base)	**E**	Assume pasta is made with fresh Everyday ingredients or packaged ingredients which have a ≥3.5 HSR and/or foods from the Essential Shopping List.<br><br>Assume pasta portion size is < max
	Packaged (tomato, mince based or cheese base)	**E**	Check portion size and HSR label on FoodSwitch database
	Freshly prepared with processed meat e.g. carbonara pasta	**O**	Assume occasional items e.g. processed meat have ≥3.5 HSR<br><br>Assume pasta portion size is < max
	Packaged pasta with processed meat	**O**	Check portion size and HSR label on FoodSwitch database
Pizza (does not contain processed meats)	Freshly prepared	**E**	Assume pizza is made on thin crust with fresh unpackaged ingredients or packaged ingredients which have ≥3.5 HSR and/or foods from the Essential Shopping List<br>Assume pizza portion size is < max
	Packaged	**E**	Check portion size and HSR label on FoodSwitch database
Pizza (contains processed meats)	Freshly prepared	**O**	Assume pizza is made with fresh Everyday ingredients or packaged ingredients which have ≥3.5 HSR and/or foods from the Essential Shopping List<br>Assume portion size of processed meat is < max portion size, e.g. ham should be <60g<br>Assume pizza portion size is < max
	Packaged	**O**	Check portion size and HSR label on FoodSwitch database
Soup	Freshly prepared	**E**	Assume soup is made with fresh Everyday ingredients or packaged ingredients which have ≥3.5 HSR and/or foods from the Essential Shopping List. Assume lean meats are used.
	Packaged	**E**	Check portion size and HSR label on FoodSwitch database
Jacket Potato	Freshly prepared	**E**	Assume jacket potato is made with fresh Everyday ingredients or packaged ingredients which have ≥3.5 HSR and/or foods from the Essential Shopping List<br>Assume portion < max
Hot chips	Packaged	**O**	Check portion size and HSR label on FoodSwitch database
Garlic bread	Freshly prepared	**O**	Assume appropriate portion size (half English muffin or pita, 1 slice of bread, 75g).<br>Assume garlic bread is made with fresh Everyday ingredients or packaged ingredients which have ≥3.5 HSR and/or foods from the Essential Shopping List

	Packaged	**O**	Check portion size and HSR label on FoodSwitch database
Pies (meat pie, vegetable pie)	Freshly prepared	**O**	If products portion size is <180g for pastry-top pies and <250g if potato-top
Pies (meat pie, vegetable pie)	Freshly prepared	**ONC**	If products portion size is >180g for pastry-top pies and >250g if potato-top OR has a HSR of <3.5 stars
Savoury pastry e.g. Sausage Roll	Freshly prepared	**O**	if savoury pastry portion size < 120g
Savoury pastry e.g. Sausage Roll	Freshly prepared	**ONC**	If savoury pastry portion size > 120g OR has a <3.5 HSR
Hot food products with no ingredient information e.g. This could include but not limited to macaroni and cheese, lasagne, meat balls, fried rice, quiche, nachos	Freshly prepared	**Assign according to appropriate category in guidelines (E or O)**	If no ingredient list available, assume home-made products are made with fresh Everyday ingredients or packaged ingredients which have ≥3.5 HSR and/or foods from the Essential Shopping List.<br><br>Assume portion size requirements are met
	Packaged	**Assign according to appropriate category in guidelines (E or O)**	Check portion size and HSR label on FoodSwitch database
Salads/sandwiches or hot prepared foods	Freshly prepared (with visible Occasional ingredients)	**ONC**	Ingredient list or visible use of :<br>-dressings made from cream, sour cream, crème fraiche<br>-chocolate nut spread and other nut spread <3.5 HSR<br>-sauces made from cream or butter<br>-regular coconut milk and cream
**Snacks**			
Fruit Cup	Freshly prepared	**E**	Assume fruit cup is made with Everyday fresh fruit with no toppings added.
	Packaged	**E**	Check portion size and HSR label on FoodSwitch database
Corn cobette	Freshly prepared/ packaged	**E**	Assume corn cobette is an Everyday vegetable and is not prepared with salt or butter.
Dips -vegetable based, yoghurt	Freshly prepared	**E**	If vegetable based: assume made with fresh Everyday vegetables and/or ingredients from the Essential Shopping List.<br><br>If dairy based: assume products used have a ≥3.5 HSR
	Packaged	**E**	Check HSR label on FoodSwitch database
Dips –cream cheese or sour cream based	Freshly prepared	**ENC**	Assume product has a <3.5 HSR
	Packaged	**ENC**	Check HSR label on FoodSwitch database
Pikelets and pancakes	Freshly prepared	**E**	Assume no toppings are added and/or toppings are from the "Buy" section of the Essentials shopping list and applied according to preparation instructions e.g. thin spread of jam
	Packaged	**E**	Check HSR label on FoodSwitch database

Popcorn	Freshly prepared	**E**	Assume popcorn is air-popped, plain (contains no salt and butter)
	Plain/ packaged	**E**	Check portion size and HSR label on FoodSwitch database, if >50g, then **ONC**
	Flavoured/ microwaved	**O**	Check portion size and HSR label on FoodSwitch database
Salty snacks	Packaged	**O**	Check HSR label on FoodSwitch database
	Packaged, >50g	**ONC**	Over allowed portion size.
	Packaged, with added confectionery (yoghurt coating, choc)	**ONC**	Occasional ingredients added
Muffins	Freshly prepared	**O**	Estimate size, assume no added Occasional ingredients
	Freshly prepared, >80g	**ONC**	Over allowed portion size
	Packaged	**ONC**	Assume ingredients have a HSR of <3.5. Also check size.
Muffin (with added confectionery: choc, icing)	Freshly prepared	**ONC**	Added confectionery<br>Assume choc muffin is <3.5 stars
	Packaged	**ONC**	Assume has <3.5 HSR
Homemade snacks such as cakes, sweet pastries	Freshly prepared	**O**	Assume item meets portion size, if ambiguous, assume ingredients have a HSR of ≥3.5
	Freshly prepared, >80g	**ONC**	Over allowed portion size
	Freshly prepared, with added confectionery: choc, icing	**ONC**	Added confectionery<br>Assume <3.5 stars
Homemade sweet biscuits	Freshly prepared	**O**	Assume item meets portion size, if ambiguous, assume ingredients have a ≥3.5 HSR
	Freshly prepared, >50g	**ONC**	Over allowed portion size
	Freshly prepared, with added confectionery: choc, icing	**ONC**	Added confectionery<br>Assume <3.5 stars
Muesli bars	Packaged	**E**	Check HSR label on FoodSwitch database
	Packaged, >50g	**ENC**	Over allowed portion size
	Packaged, with added confectionery (icing, choc, yoghurt coatings)	**O**	Added confectionery<br>Assume <3.5 stars
Confectionery	Packaged	**O**	Assume confectionery is <3.5 stars
	Packaged, if >50g	**ONC**	Over allowed portion size
Jelly	Freshly prepared	**O**	Assume jelly is <3.5 stars
	Packaged	**O**	Assume jelly is <3.5 stars
Jelly sticks, tubes, wobbli or pouch are 'juice jellies therefore confectionary'	Confectionary	**O**	Assume sticks, tubes, wobbli or pouch are 'juice jellies therefore confectionary'
**Dairy foods**			

Yoghurt, cheese	Packaged	**E**	Check HSR label on FoodSwitch database
	Packaged, with added confectionery	**O**	Assume all have a <3.5 HSR
**Drinks**			
Water	Packaged	**E**	Assume has a ≥3.5 HSR
Plain milk e.g. cow and plant based milks	Packaged	**E**	Assume plain milk has a ≥3.5 HSR.
Flavoured milk	Freshly prepared	**E**	If ambiguous, assume portion size < max and no more than 1 level tablespoon of milk flavouring or powder e.g. Milo, hot chocolate
	Packaged	**E**	If ambiguous, assume portion size < max and has a HSR of 3.5 stars and above
Milkshakes/ smoothies	Freshly prepared	**E**	If ambiguous, assume portion size < max and is made with ingredients that have a ≥3.5 HSR
	Packaged	**E**	If ambiguous, assume portion size < max and is made with ingredients that have a ≥3.5 HSR
Juice (poppers, pop-tops, frozen juice/ frozen crushed ice drinks)	Packaged	**E**	Check HSR label on FoodSwitch database
Diet soft drinks	Packaged	**O**	Note portion size
**Frozen ice snacks**			
Ice block	Packaged	**O**	Assume portion size <125mL and has a HSR of <3.5 stars
Slushies	Packaged	**E**	If ambiguous, assume 99% fruit juice, portion size < max and HSR of 3.5 stars and above
Ice cream	Packaged	**O**	If ambiguous, assume portion size >125mL and has a HSR of <3.5 stars
Milky bites (frozen milk)	Freshly prepared	**E**	If ambiguous, assume this is low fat flavoured milk with HSR of 3.5 stars and above
Frozen yoghurt	Packaged	**O**	If ambiguous, assume portion size >125mL and has a HSR of 3.5 stars and above.
Ice cups e.g. Made with cordial	Freshly prepared	**O**	If portion size of ice cups > max (125g) and not made with 99% fruit juice.
**Condiments – spreads and sauces**			
Sauces – tomato, BBQ and soy sauce (pg.18)	Packaged	**Compliant**	Assume all sauces are on the 'buy section' of the shopping list and soy and satay sauces are salt reduced<br>**Note**: whilst this is the assumption most canteens will not sell salt reduced sauce
Margarine (pg. 17)	Packaged	**Compliant**	Assume margarine has a ≥3.5 HSR.
